# A bi-hemispheric neuronal network model of the cerebellum with spontaneous climbing fiber firing produces asymmetrical motor learning during robot control

**DOI:** 10.3389/fncir.2014.00131

**Published:** 2014-11-05

**Authors:** Ruben-Dario Pinzon-Morales, Yutaka Hirata

**Affiliations:** ^1^Neural Cybernetics Laboratory, Department of Computer Science, Chubu UniversityKasugai, Japan; ^2^Department of Robotic Science and Technology, Chubu UniversityKasugai, Japan

**Keywords:** cerebellum, asymmetrical motor learning, cerebellar hemispheres, adaptive robot control, climbing fiber, modeling

## Abstract

To acquire and maintain precise movement controls over a lifespan, changes in the physical and physiological characteristics of muscles must be compensated for adaptively. The cerebellum plays a crucial role in such adaptation. Changes in muscle characteristics are not always symmetrical. For example, it is unlikely that muscles that bend and straighten a joint will change to the same degree. Thus, different (i.e., asymmetrical) adaptation is required for bending and straightening motions. To date, little is known about the role of the cerebellum in asymmetrical adaptation. Here, we investigate the cerebellar mechanisms required for asymmetrical adaptation using a bi-hemispheric cerebellar neuronal network model (biCNN). The bi-hemispheric structure is inspired by the observation that lesioning one hemisphere reduces motor performance asymmetrically. The biCNN model was constructed to run in real-time and used to control an unstable two-wheeled balancing robot. The load of the robot and its environment were modified to create asymmetrical perturbations. Plasticity at parallel fiber-Purkinje cell synapses in the biCNN model was driven by error signal in the climbing fiber (cf) input. This cf input was configured to increase and decrease its firing rate from its spontaneous firing rate (approximately 1 Hz) with sensory errors in the preferred and non-preferred direction of each hemisphere, as demonstrated in the monkey cerebellum. Our results showed that asymmetrical conditions were successfully handled by the biCNN model, in contrast to a single hemisphere model or a classical non-adaptive proportional and derivative controller. Further, the spontaneous activity of the cf, while relatively small, was critical for balancing the contribution of each cerebellar hemisphere to the overall motor command sent to the robot. Eliminating the spontaneous activity compromised the asymmetrical learning capabilities of the biCNN model. Thus, we conclude that a bi-hemispheric structure and adequate spontaneous activity of cf inputs are critical for cerebellar asymmetrical motor learning.

## 1. Introduction

Development, aging, and injuries are common conditions that prevent the neural centers governing the muscles from being rigid and hard-wired. Thus, a key feature of these centers is adaptation. The cerebellum is one example of a neural center where adaptation is crucial. The cerebellum is involved in cognition (Thach, 1998; Manto et al., 2013; Overwalle et al., 2014), motor learning, and coordination (Thach, 1996; Highstein et al., 2005; Spencer et al., 2005; Ito, 2011; Manto et al., 2012). Adaptation in the cerebellum has been widely studied in eye movements such as smooth pursuit (Belknap and Noda, 1987; Stone and Lisberger, 1990), the vestibuloocular reflex (VOR) (Lisberger et al., 1994; Ito, 1998; Hirata and Highstein, 2001; Blazquez et al., 2003; Broussard and Kassardjian, 2004), and saccades (Hopp and Fuchs, 2004; Kojima et al., 2010) because these adaptations can be evoked easily under experimental conditions. For example, VOR gain, defined as eye velocity divided by head velocity during head turn, can be tuned up using a visual-vestibular mismatch stimulus (Melvill Jones et al., 1988; Paige and Sargent, 1991; Kassardjian et al., 2005; Anzai et al., 2010). Under normal circumstances, the cerebellum exerts symmetrical control over the muscle plant of the eyes (Demer, 1992); however, when the muscle plant is changed by aging, lesions, or asymmetrical optics, the cerebellum must compensate for the abnormal conditions by adapting asymmetrically (Marti et al., 2006). Asymmetrical adaptation can also be elicited in the laboratory. The vertical VOR gain can be increased in the up direction (i.e., downward head turn) and decreased in the down direction (i.e., upward head turn) simultaneously in monkeys (Hirata et al., 2002; Ushio et al., 2011) and humans (Marti et al., 2006). In goldfish, the same type of asymmetrical VOR adaptation can be induced in the horizontal system (Yoshikawa and Hirata, 2006). Saccades have also been shown to be asymmetrically tunable (Snow et al., 1985; Erkelens et al., 1989; Lemij and Collewijn, 1991; Hopp and Fuchs, 2004).

Despite these lines of experimental evidence, the loci and the neural mechanisms underlying asymmetrical adaptation are a matter of debate. Several possible loci have been proposed involved in VOR asymmetrical adaptation, including direction-sensitive and non-linear firing responses of gaze-velocity floccular Purkinje cells (Marti et al., 2006), or the floccular target neurons in the vestibular nucleus and the dorsal Y group (Blazquez et al., 2000; Hirata et al., 2002). These loci constitute partially independent mechanisms for adaptive control of vertical VOR gain, whereas the bilateral pool of motor neurons innervating the muscles of the eye has been proposed to be the locus of dis-conjugate adaptation in saccades (Kapoula et al., 1996). A different hypothesis proposes that the asymmetrical mechanisms are closely related to the bi-hemispheric structure of the cerebellum (Choi et al., 2008; Ohki et al., 2009; Panouilleres et al., 2012). Lesions to the left cerebellar hemisphere lobule H-VII of the monkey significantly impair motor performance in the ipsiversive direction but also to a less degree in the contraversive direction during smooth pursuit (Ohki et al., 2009). In a similar way, the adaptation of postsaccadic smooth pursuit velocity affects the ipsiversive direction (Ohki et al., 2009). Ipsilesional saccadic adaptation was significantly reduced following unilateral cerebellar hemisphere infarctions in humans (Choi et al., 2008). These results suggest that a bi-hemispherical structure is necessary for asymmetrical motor adaptation and that the hemispheres are not completely independent.

Climbing fiber (cf) input is an important mechanistic link between the two cerebellar hemispheres. Cf input has been proposed to carry the error signal required for long-term changes in the sensitivity of Purkinje cells to specific inputs from mossy fibers (Marr, 1969; Albus, 1971; Ito et al., 1982), for the more immediate and short-term effects on the simple spikes of Purkinje cells (Mano, 1974; Medina and Lisberger, 2008), and for the rapid and strong (phasic) override commands to Purkinje cells (Llinas, 2011). Monkey experiments during horizontal VOR gain adaptation showed that the cf input encoded information about the amount and direction of the error (Hirata et al., 2006, 2007). The major type of cf input in the left hemisphere increased its firing rate with ipsidirectional retinal error, whereas it decreased its firing rate below its spontaneous firing rate (approximately 1 Hz), with contradirectional retinal error. The cf input also showed similar characteristics during smooth pursuit experiments in monkeys (Stone and Lisberger, 1990). Therefore, assuming that cf input drives motor learning in the cerebellar circuit, adaptation in one hemisphere takes into account both the error information in its preferred direction and its non-preferred direction (i.e., in the preferred direction of the contralateral hemisphere).

Examination of the role of the cerebellar hemispheres and the cf input during asymmetrical motor adaptation is relevant not only for deepening our understanding of the biological system but also for applications involving cerebellar models in various engineering problems. Therefore, we designed a realistic neuronal network model of the cerebellum to investigate the mechanisms behind asymmetrical adaptation. We include a bi-hemispheric neural network model of the cerebellum (biCNN) and a realistic cf input with spontaneous activity. We chose a control engineering framework to test the capacity of the biCNN model. We evaluate the biCNN model by allowing it to control a two-wheel balancing robot in real-time, which allowed us to reproduce conditions in which asymmetrical adaptation is required. Specifically, we addressed (1) control performance during a symmetrical perturbation (i.e., a load on the top and center of the vertical axis of the robot), (2) control performance during two different asymmetrical perturbations (i.e., load on top and off-center to the front or back of the robot and declining/inclining the platform where the robot moves), and (3) the role of spontaneous activity of the cf input in the real time robot experiments.

## 2. Materials and methods

### 2.1. Overview of the bi-hemispherical neuronal network model of the cerebellum (biCNN)

**Figure 1:**
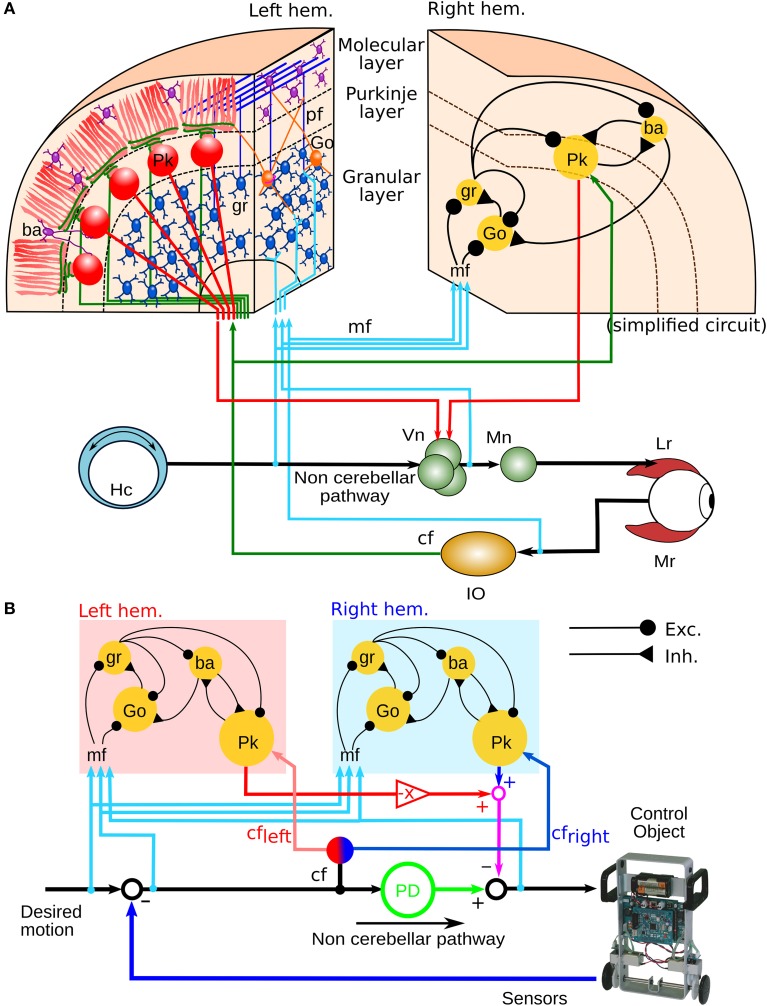
**Neural circuit of the horizontal VOR and bi-hemispheric neural network model of the cerebellum (biCNN model). (A)** The neural circuit of the horizontal VOR. IO, inferior olive; Vn, vestibular nucleus; Go, Golgi cell; gr, granular cell; Lr, lateral rectus muscle of the eye; Mr, medial rectus muscle of the eye; mf, mossy fiber; Mn, motor neuron; Ba, basket/stellate cell; pf, parallel fibers; Hc, horizontal canal. **(B)** The structure of the biCNN model, including a proportional and derivative (PD) feedback controller that represents the non-cerebellar pathway depicted in **(A)**.

Previously, we configured a physio-anatomically inspired cerebellar neuronal network (CNN) controller based on the neuronal microcircuits of the cerebellar cortex (Figure 1A) that are involved in horizontal VOR (flocculus: FL) (Pinzon-Morales and Hirata, in review). The CNN comprises cell types whose physiological and anatomical properties are well understood (Ito, 2011). These include granular (gr) cells, Golgi (Go) cells, basket and stellate (here both are referred to as ba) cells, and Purkinje (Pk) cells. The CNN receives two types of inputs carried by mossy fibers (mfs) and a climbing fiber (cf), as in the real cerebellum. Connectivity in the CNN includes an excitatory projection from mfs to gr and Go and from gr to ba and Go, an inhibitory feedback loop between gr and Go, and a feed-forward inhibitory loop between ba and Pk (Pinzon-Morales and Hirata, in review). The bi-hemispheric neuronal network model of the cerebellum (biCNN model) (Figure 1B) comprises two CNNs corresponding to the left and right cerebellar hemispheres. The two CNNs were updated from our previous work by including three improvements. First, we included inhibitory connections from ba to Go cells, and mutual inhibitory synapses between ba and Pk cells, which have been characterized electrophysiologically (O'Donoghue et al., 1989; Dumoulin et al., 2001; Maex and Schutter, 2005). Second, we increased the number of neuron models to account for the number of neurons found in a cube of the cerebellar cortex with sides of 100μm (see below Section 2.5) (Solinas et al., 2010). Third, we included plasticity at synapses between Pk and the vestibular nucleus (Vn), which were proposed to be the locus of memory consolidation after motor learning (Masuda and Amari, 2008; Yamazaki and Nagao, 2012) (see below Section 2.4). The hemispheres share the same mf inputs carrying the desired motion signals (vestibular afferents), efference copy of motor commands, and sensory error signals (desired trajectory – actual trajectory) (Noda, 1986; Hirata and Highstein, 2001; Blazquez et al., 2003; Huang et al., 2013). Cf inputs with different sensory error configurations reach each hemisphere (see below Section 2.2). The output of the left hemisphere is inverted (Pk firing rates in the interval [−1 0]), added to the right hemisphere output (Pk firing rates in the interval [0 1]) and sent to the Vn. The other input to the Vn, the non-cerebellar pathway (Figure 1A), is generated by the output of a proportional and derivative (PD) controller that is a feedback controller widely used in industry and other applications (Figure 1B). The parameters of the PD controller (proportional gain *K_p_* and derivative gain *K_d_*) were designed following optimal settings for automatic controllers (Ziegler and Nichols, 1942), so that the PD controller alone can stably operate the robot during a simple task [θ_*ref*_(*t*) = 0, ϕ_*ref*_(*t*) = π sin(2π0.1*t*), where θ and ϕ are the robot's body and wheel angle control variables, respectively, (see below Section 2.7)]. The Vn generates the motor command to the robot as the arithmetic subtraction of the hemispheric activities (firing rate in the range [−1 1]) from the PD output (in the range [−1 1] A). This model is open source and available via contact with the authors.

### 2.2. Climbing fiber input

**Figure 2:**
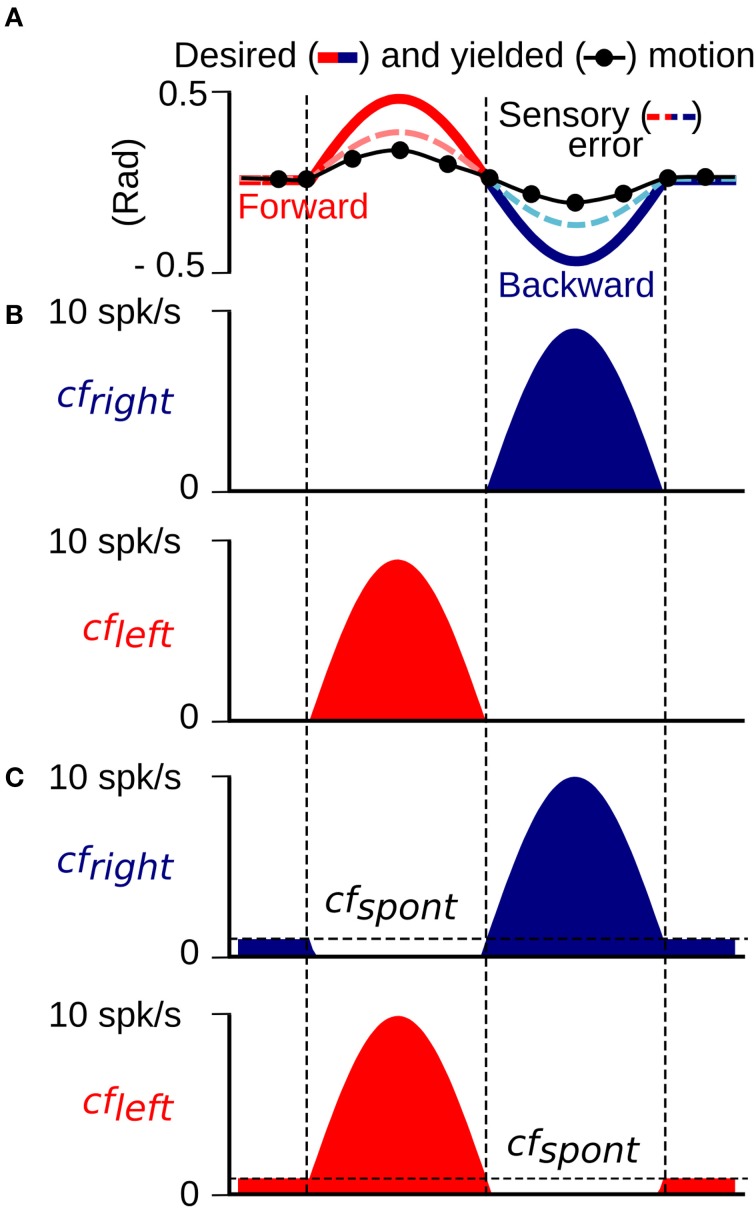
**Construction of the climbing fiber (sensory error) input to the left (*cf*_*left*_) and right (*cf*_*right*_) hemisphere. (A)** An example of cf generated as the difference between the desired and yielded motion. **(B)** *cf*_*left*_ and *cf*_*right*_ inputs without a spontaneous firing rate created from the positive and negative half-waves of the cf input in **(A)**. **(C)** *cf*_*left*_ and *cf*_*right*_ inputs with a spontaneous firing rate determined by the threshold *cf*_*spont*_.

The cf input to the biCNN model, which has been proposed to carry the error information required for driving plasticity at parallel fiber (pf)-Pk synapses as a basis for motor learning (Marr, 1969; Ito, 2013), is calculated in the model from the difference between the desired and actual motion (Hirata et al., 2002; Ito, 2011, 2013; Pinzon-Morales and Hirata, in review) (Figure 1B). The cf input carries position and velocity error components in kinematic coordinates (expressed in angle units, rads). Our previous work (Pinzon-Morales and Hirata, in review) demonstrated that the cf conveys error in motor coordinates [a copy of the motor command aimed at minimizing the sensory error (Kawato and Gomi, 1992; Kawato, 1999)] or a combination of errors in motor and sensor coordinates (Kitazawa et al., 1998), and can drive adequate plasticity in the CNN model (Pinzon-Morales and Hirata, in review). We employ cf to carry sensory error in the current application because it yielded the highest control performance (Pinzon-Morales and Hirata, in review). Sensory error (shown in Figure 2A) was split into forward and backward errors (Figure 2B) (sensory error > 0, and sensory error < 0, respectively) and spontaneous activity (*cf*_*spont*_ = 0.05) was added (Figure 2). A reduction of the cf activity below its spontaneous rate (approximately 1 Hz) has been shown to correlate with sensory errors produced in the non-preferred direction (Hirata et al., 2006, 2007). Thus, each hemisphere was configured to receive a cf input carrying information from mainly one direction of robot motion, i.e., the left hemisphere received mainly forward sensory error (Figure 2B, *cf*_*left*_), whereas the right hemisphere receives backward sensory error (Figure 2B, *cf*_*right*_). In this way, the cf increases its firing rate above the *cf*_*spont*_ level with erroneous motion in the preferred direction, whereas, erroneous motions in the non-preferred direction causes a reduction of the cf firing rate below the value *cf*_*spont*_.

### 2.3. pf-Pk plasticity

The biCNN model includes plasticity at two different loci, the pf-Pk and Pk-Vn synapses. In contrast, our previous CNN included plasticity at only the pf-Pk synapses (Pinzon-Morales and Hirata, in review). The pf-Pk synapses are the loci where motor learning has been classically proposed to be stored (Boyden et al., 2004; Ito, 2011; Marquez-Ruiz and Cheron, 2012) via two mechanisms: long-term depression (LTD) and long-term potentiation (LTP). LTD is expressed as a decrease in the synaptic efficacy that occurs with concurrent pf and cf activity, whereas LTP is an increase in the synaptic efficacy driven by sustained pf activity in the absence of cf input. The present model implements plasticity at pk-Pf as follows:

(1) $$\Delta W_{pf_{i}-Pk_{j}}(t)=\left\{ \begin{array}{l} \;\gamma_{\text{LTD}}\;cf(t)pf_{i}(t)\text{ if }cf(t)>cf_{spont}\\ \;\gamma_{\text{LTP}}pf_{i}(t)\text{ if}\;cf(t)<cf_{spont} \end{array}\right.$$

where Δ*W_pf_i_ − Pk_j__*(*t*) is the change in the synaptic weight between the *i*-th pf and the target *j*-th Pk, *cf*(*t*) is the error signal reaching each hemisphere (depicted in Figure 2), *pf_i_*(*t*) is the firing rate of the *i*-th pf (in the range [0 1]), and γ_LTD_ = −4 × 10^−6^ and γ_LTP_ = 0.3 × 10^−6^ are the learning rates for LTD and LTP, respectively. The threshold value *cf*_*spont*_ = 0.05 represents the spontaneous activity in the cf.

### 2.4. Pk-Vn plasticity

Plasticity at the Pk-Vn synapses has been proposed to depend on the activity of the Vn and Pk (Masuda and Amari, 2008; Yamazaki and Nagao, 2012; Garrido Alcazar et al., 2013). In the present model, this learning rule was implemented as follows:

(2) $$\Delta W_{Pk_{j}-Vn}(t)=\gamma Vn(t)(Pk_{i}(t)-0.5)$$

where Δ*W_Pk_j_−Vn_*(*t*) is the change in the synaptic weight between the *j*-th Pk and the Vn, *Vn*(*t*) is the activity of the Vn (in the range [−1 1]), *Pk*(*t*) is the firing rate of the Pk (in the range [0 1]), and γ = 1 × 10^−4^ is the learning rate. Note that the constant 0.5 subtracted from *Pk*(*t*) is required to convert the firing rate to the same dominion of the Vn ([−1 1]). In this algorithm, when the sign of (*Pk*(*t*) − 0.5) and *Vn*(*t*) are different, the synaptic weights are decreased (LTD), whereas when their signs are the same, the weights are increased (LTP).

### 2.5. Construction of the biCNN model

Two CNNs of equal characteristics were configured to construct the biCNN model, as described above. Here, we describe one hemisphere in detail. Based on the reported density of gr cells, 4 × 10^6^/mm^3^ (Solinas et al., 2010), our network includes 4096 gr cell models, corresponding to the volume of a cube of the cerebellum with an edge length of 100 μm. Similarly, the density of ba has been reported to be 32 × 10^3^/mm^3^ (Solinas et al., 2010); thus, we included 274 ba in the neuronal network. Considering the ratio of ba cells to Pk cells (~200:1, Ito, 2011), and the ratio of Go to gr cells (~1:2000, Maex and Schutter, 1998), 15 Pk and 28 Go cells were included in the neuronal network. In total, there were 4694 neuron models in each hemisphere, and 9388 neurons in the biCNN model.

**Figure 3:**
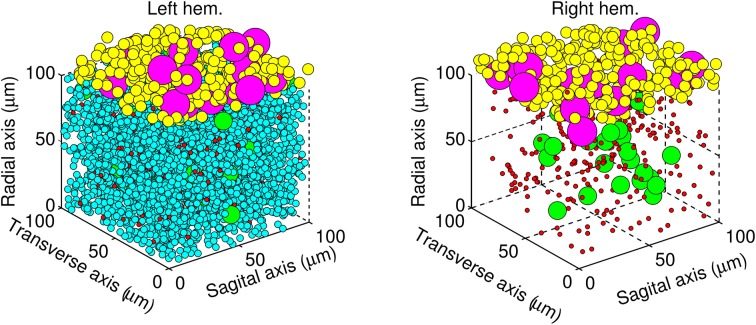
**Architecture of the biCNN model showing the left hemisphere (left panel) with all the neurons, and for the sake of visualization, the right hemisphere (right panel) lacking gr cells**. Granular cells (cyan), Golgi cells (green), mossy fibers glomeruli (red), basket cells (yellow), and Purkinje cells (magenta) are shown.

The next step was to place each neuron inside a volume of cerebellar tissue represented by a cube of edge length 100 μm. The biCNN model was constructed in two layers. The granular layer was placed inside the cube and the molecular/Purkinje layer outside, at the top of the cube. Go, gr, and mf glomeruli were placed in the granular layer, whereas Pk and ba cells were placed in the molecular/Purkinje layer. The relative size of each type of neuron was included as a constraint in the random allocation procedure to forbid neurons from occupying the same space. Figure 3 shows the resulting allocation of the neurons in both hemispheres.

The connections of each neuron were built using a nearest-neighborhood rule and the convergence/divergence ratios of each cell type (Table 1), which followed the reported anatomical ratios as closely as possible for the given number of neurons in our model. For instance, for a gr cell that receives four different mfs and four different Go inputs, our procedure connected the four closest mfs and Go cells. This procedure, along with the random allocation of neurons inside the cube, ensured the uniqueness of each hemisphere while conserving the general characteristics of the cerebellar microcircuit. Additionally, our procedure provided the network with spatial information that has been shown to encode relevant clues about how information is processed in the cerebellum (Solinas et al., 2010). In total, there are 110,300 synapses in each hemisphere of the biCNN model. Random synaptic weights were extracted from a normal distribution (μ = 0.9 and σ = 0.1 ∈ [0.8, 1]) and multiplied by a normalizing constant (*d*) that was cell dependent. *d* is determined as the inverse of the number of inputs of the same nature (excitatory or inhibitory) of each cell. For instance, a Go cell with seven mf excitatory inputs had the normalizing constant *d* = 1/7 for the synaptic weights of the mf inputs, *d* = 1/1639 for the 1639 synaptic inputs of the excitatory gr inputs, and *d* = 1/3 for the three inhibitory ba inputs.

**Table 1:** **Convergence and divergence synaptic ratio of the biCNN model**.

	**Num.cells**	**Divergence**	**Convergence**	
Mossy fibers (mf)	562			
Golgi (Go)	56			
Granular (gr)	8192			
Basket/Stellate (ba)	548			
Purkinje (Pk)	30			
mf→ gr		1:59	4:1	Solinas et al., 2010; Ito, 2011
mf→ Go		1:7	66:1	Solinas et al., 2010; Ito, 2011
gr (pf)→ Go		1:12	1639:1	Solinas et al., 2010; Ito, 2011
Go→ Gr		1:586	4:1	Solinas et al., 2010; Ito, 2011
gr (pf)→ Ba		1:3	41:1	Maex and Schutter, 1998; Ito, 2011
gr (pf)→ Pk		1:4	1024:1	Ito, 2011
ba→ Pk		1:7	110:1	Solinas et al., 2010
Pk→ Ba		1:55	3:1	Schilling et al., 2008
Ba→ Go		1:3	28:1	Dieudonne and Dumoulin, 2000

*Number of mf inputs to the cerebellar model changes with the control plant*.

### 2.6. Address event representation for real time implementation of the biCNN model

**Figure 4:**
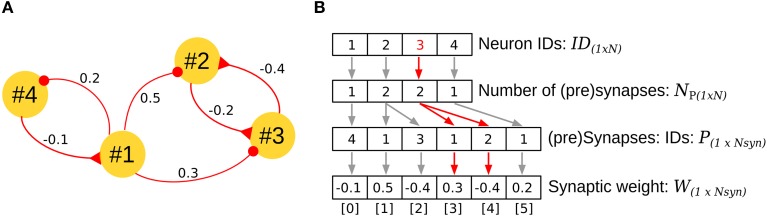
**Address Event Representation (AER) for implementation of the biCNN model. (A)** Example of AER for a network with four neurons. **(B)** Vectors required for representing the network in **(A)** by using AER. The specific case for neuron #3 is shown in red. *N* stands for the number of neurons (*N* = 4 in the example), and *N*_*syn*_, the number of synapses in the network (*N*_*syn*_ = 6 in the example).

Address Event Representation (AER) is a communication technique for sparse networks and has been successfully extrapolated to neural networks (Johansson and Lansner, 2007). In AER, four vectors are required to describe the network architecture (Figure 4B). The first vector *ID* encodes the neurons in the network, assigning each a unique ID. The second vector *N*_*P*_ stores the number of pre-synapses for each neuron, and the third and fourth vectors *P* and *W* encode the IDs of the pre-synaptic neurons and the corresponding synaptic weights in a stacked, ordered way. For example, in Figure 4A, neuron #3 is contacted by two neurons (#2, #1); this information is clearly observed in the third element of vectors *ID*[2] = 3, *N*_*P*_[2] = 2 (red arrows Figure 4B). By accumulating the number of synapses for the neurons that precede the neuron #3 (neuron #2, 2 pre-synapses, neuron #1, 1 pre-synapse), the index for the pre-synapses of neuron #3 can be read in the vectors *P*[3] = 1, and *P*[4] = 2, with their respective weights in *W*[3] = 0.3, and *W*[4] = −0.4.

The AER representation is compact, efficient in terms of memory consumption, and convenient for implementation using dataflow programming frameworks such as LabVIEW (National Instrument, Austin, TX). Efficient implementation can be achieved by a careful selection of the neuron IDs according to the flow of data in the network. This combination of software and architecture allows the construction of larger neuronal networks that can run in real time (20 k neurons with 240 k synapses: real time 10 ms, execution time 2.2 ms on a Windows computer 4 × 3.33 Ghz Intel Core-i7 processor, memory: 16 GB running LabVIEW 2010. For different setups and sampling times, see Pinzon-Morales and Hirata, 2014).

### 2.7. Control object and experimental protocol

**Figure 5:**
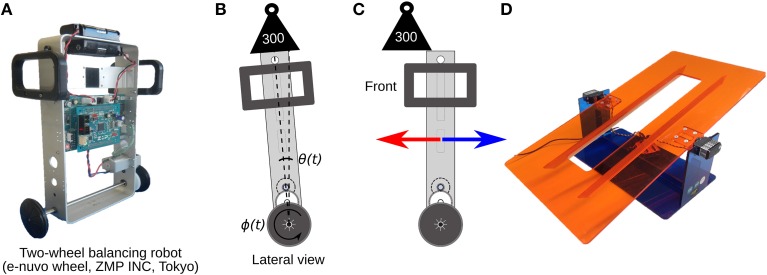
**Control plant and symmetrical/asymmetrical perturbations. (A)** Two wheel balancing robot. **(B)** Cartoon representing the lateral view of the robot during the symmetrical perturbation by adding a load on-center of the robot's vertical axis. θ(*t*) and ϕ(*t*) are the robot's body and wheel position control variables irrespectively. **(C)** Asymmetrical perturbation by adding an external load off-center of the vertical axis of the robot. **(D)** Asymmetrical perturbation by changing the angle of the platform where the robot moves.

The two-wheel balancing robot (e-nuvo wheel, ZMP INC, Tokyo) (Figure 5) is an inverted pendulum system widely used in control engineering for testing control strategies, because of its highly unstable dynamics. The robot is considered one of the most challenging control plants (Li et al., 2013). The robot is equipped with a set of sensors including a motor encoder and a gyroscope, which provide wheel angle (ϕ(*t*)) and body tilt angle (θ(*t*)), respectively. The robot is also equipped with a USART chip to allow serial communication with the computer on which the biCNN model is implemented at sampling period *T*_*s*_ = 10 ms, which is the same time interval used in the present biCNN model. The motion of the robot is driven by a single DC motor connected to the 2 wheels, which share the same shaft. The mf inputs for this control object carry the following signals: (1) desired wheel angle ϕ_*ref*_(*t*), (2) desired wheel angular velocity $\dot{\phi }$_*ref*_(*t*), (3) body tilt angle error θ_*e*_(*t*) = θ_*ref*_(*t*) − θ(*t*), where θ_*ref*_(*t*) is the desired body tilt angle, (4) body tilt angular velocity error $\dot{\theta }$_*e*_(*t*) = $\dot{\theta }$_*ref*_(*t*) − $\dot{\theta }$(*t*), where $\dot{\theta }$_*ref*_(*t*) is the desired body tilt angle, (5) wheel angle error ϕ_*e*_(*t*) = ϕ_*ref*_(*t*) − ϕ(*t*), (6) wheel angular velocity error $\dot{\phi }$_*e*_(*t*) = $\dot{\phi }$_*ref*_(*t*) − $\dot{\phi }$(*t*), and (7) efference copy of motor command. The desired body tilt angle θ_*ref*_(*t*) and velocity $\dot{\theta }$_*ref*_(*t*) were set to zero radians, so that the robot is commanded to remain vertical while following the desired wheel angle trajectory, which was set to a sinusoidal motion ϕ_*ref*_(*t*) = π sin(2π0.25*t*). These seven mfs were repeated 81 times to generate the 562 mfs required in the biCNN model. Perturbations to the robot, symmetrical and asymmetrical, were created by placing an external load (300 g, 50% of robot's mass) on the top and center of the vertical axis of the robot (symmetrical load depicted in Figure 5B), off-center on the front/back (asymmetrical load depicted in Figure 5C), or by changing the angle of the platform on which the robot was moving (depicted in Figure 5D).

## 3. Results

### 3.1. Symmetrical motor control scenario

**Figure 6:**
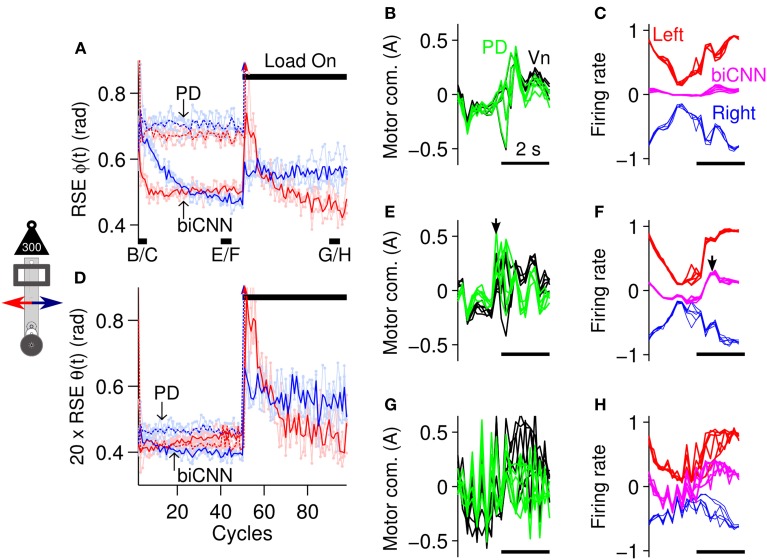
**biCNN model performance during control of the robot with external symmetrical perturbation. (A)** Average control performance (*N* = 6) in terms of RSE of ϕ(*t*) for the forward (red lines) and backward (blue lines) motions of the robot. Light red and blue lines show raw RSE performance for the repetitions (*N* = 6) of the experiment. An external load (300 g) was placed on-center of the robot vertical axis at cycle #50. The performance obtained by using only the PD controller is shown labeled “PD,” whereas the performance attained with the biCNN model is labeled “biCNN.” **(B)** Five cycles of the PD (green lines) and Vn (black lines) outputs at the beginning of the experiment (cycles #2-6, black line shown in **(A)** with label B/C) are superimposed and aligned. **(C)** The same five cycles in **(B)** for the left (red lines) and right (blue lines) hemispheres, and the biCNN model (magenta lines) firing rates. For the sake of comparison, the outputs of the hemispheres and their sum are shown inverted. **(D)** RSE of θ(*t*) in the same format as **(A)**. **(E)** Five cycles of the PD and Vn outputs before the perturbation [cycles #40-45, black line shown in **(A)** with label E/F] in the same format as **(B)**. **(F)** In the same format as **(C)** for the cycles used in **(E)**. The black arrows **(E,F)** show that the biCNN model output lags compared with the PD and Vn output. **(G)** In the same format as **(B,E)** for cycles at the end of the experiment [cycles #90-95, black line shown in **(A)** with label G/H]. **(H)** In the same format as **(C,F)** for the cycles used in **(G)**. Note that the PD could not control the robot after the perturbation. Color notation follows the same format as Figure 1B.

Before exploring the asymmetrical capabilities of the biCNN model, in this section, a symmetrical control scenario was tested to contextualize the need for a bi-hemispherical structure. The control scenario employed consisted of 100 cycles of the sinusoidal desired motion [ϕ_*ref*_(*t*) = π sin(2π0.25*t*), θ_*ref*_(*t*) = 0] for the robot. Control of the robot remained undisturbed until cycle #50, when an external symmetrical perturbation, a load of 300 g or 50% of the robot's mass, was placed on-center of the vertical axis of the robot (depicted in Figure 5B), thus causing a close-to-symmetrical perturbation to the robot. Figure 6 summarizes the control performance attained and shows that the biCNN model was able to control the robot and compensate for the external perturbation. The control performance, measured as the root mean square error (RSE) of ϕ(*t*) (Figure 6A) and θ(*t*) (Figure 6D) of the forward (red lines) and backward (blue lines) motions of the robot [positive and negative half-rectified waves of ϕ_*ref*_(*t* + 10 sec), respectively], shows that during the initial 20 cycles of the desired motion, the biCNN model adapted to reduce the RSE on each of the robot's control variables [highly marked in the RSE of ϕ(*t*)]. The benefit of using the biCNN model in this control scenario is clearly recognized by comparing the RSE with the one achieved by using only the PD controller (Figures 6A,D, lines labeled as “PD”). The average improvement (among a total of 6 repetitions of the experiment) was 0.229 ± 0.010 rad (forward), 0.277 ± 0.016 rad (backward) and (0.0348 ± 0.010)/20 rad (forward), (0.0809 ± 0.022)/20 rad (backward) for ϕ(*t*) and θ(*t*)), respectively. Furthermore, the PD controller alone always failed to control the robot after the external load was added (Figure 6A PD arrows toward infinity after cycle #50). In contrast, Figures 6A,D show that after the external load was placed on-center along the robot's vertical axis (cycle #50-100), the biCNN model re-adapted its output to maintain adequate control of the robot and reduced the RSE. In this control scenario, the RSE of θ(*t*) was more affected than ϕ(*t*) because the extra inertia produced by the perturbation made it more difficult to achieve vertical alignment of the robot's body [i.e., θ(*t*) ≠ 0]. In addition, the RSE performances shown in Figures 6A,D (cycles #1-50) imply that the forward (red lines) and backward (blue lines) motions of the robot are inherently different. These differences are mainly due to the intrinsic asymmetries of the mechanics of the robot, which were more accentuated when the perturbation was added (Figures 6A,D, cycles #50-100).

The changes to the biCNN model output produced by the adaptation in the left and right hemispheres are shown in Figures 6C,F,H (magenta, red, and blue lines, respectively). These figures show five superimposed cycles of the firing rate of the biCNN model, left and right hemisphere, at the beginning (Figure 6C, cycles #2-6, black line shown in Figure 6A with label a), before the perturbation (Figure 6F, cycles #40-45, black line shown in Figure 6A with label b), and at the end of the experiment (Figure 6H, cycles #90-95, black line shown in Figure 6A with label c). For the sake of comparison, Figures 6B,E,G show the corresponding five cycles of the PD (green lines) and Vn (black lines) outputs. At the beginning, when the Pk cells were untrained, the default hemisphere outputs canceled each other out, resulting in a small biCNN output (Figure 6C). During this period, the non-cerebellar pathway (i.e., the PD output) was the output contributing the most to the Vn (Figure 6B, PD and Vn lines are almost equal). Then, by cycles #40-45, plasticity at the pf-Pk synapses driven by the cf input caused different modulations of the firing rate of each hemisphere (i.e., average firing rate of the Pk cells), reflecting the intrinsic differences of the forward and backward motions of the robot (Figure 6F, red and blue lines). The addition of these two outputs produced the biCNN model output (Figure 6F, magenta lines), which contributed with the PD controller (Figure 6E, green line) to the Vn output, i.e., the motor command sent to the robot. Note that the PD and the Vn outputs were no longer equal, meaning that the biCNN model was contributing to the Vn output. The apparent phase lag between the biCNN model and the PD output (Figures 6E,F, black arrows) is a direct cause of using a sensory error signal as the cf input in our model (Pinzon-Morales and Hirata, in review). This phase difference suggests that the biCNN model output adapted to cooperate with the non-cerebellar pathway input to the Vn (i.e., the PD output) to generate the motor command to the robot (Pinzon-Morales and Hirata, in review). At the end of the experiment (Figures 6G,H), the PD output was severely affected by the external perturbation, whereas the biCNN model output increased its amplitude by 60% (in comparison with the value before the perturbation). Interestingly, this net increase in the biCNN model output was caused by changes in the shape of the outputs of the hemispheres, which reflected a reduction of their peak-to-peak firing rate (17 and 23% left and right hemispheres, with respect to the values before the perturbation), reflecting the LTD occurring with the increase in the error signal produced by the external load. As a result of the adaptation in the biCNN model, the motor command remained adequate to compensate for the extra load and reduced the RSE (Figures 6A,D, cycles #50-100).

In the present close-to-symmetrical control scenario, the biCNN model hemispheres successfully learned the control sequences that reduced the error signal in the cf input and successfully controlled the robot at different frequencies of the desired motion (Supplementary Figure 2). However, a bi-hemispheric structure is not essential for this control scenario, because a uni-hemispheric CNN is also able to control the robot and compensate for the external perturbation when the load is placed on-center of the robot (Pinzon-Morales and Hirata, in review). Thus, the performance attained with the biCNN model was not different from the one attained with a uni-hemispheric configuration in this control scenario. However, when asymmetrical loads are considered, the uni-hemispheric CNN model is not able to control the robot (Supplementary Figure 1). In the next section the capability of the biCNN model to handle asymmetrical conditions is tested.

### 3.2. Asymmetrical motor control scenario

**Figure 7:**
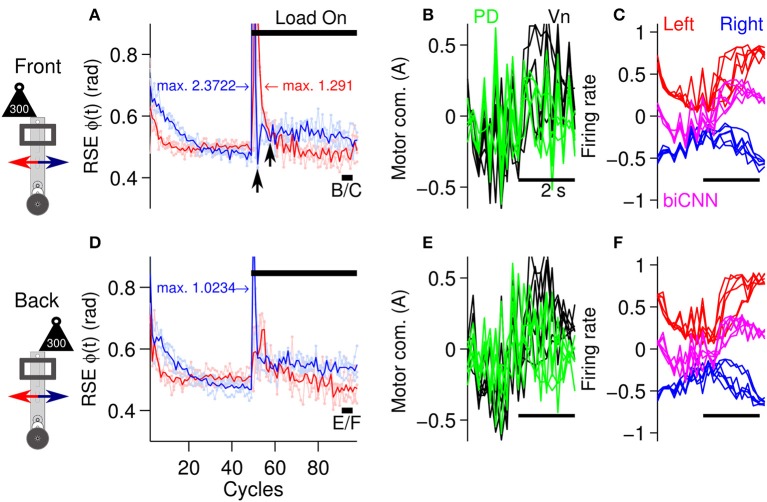
**Performance of the robot with the biCNN model under asymmetrical conditions with an external load. (A)** Control performance in terms of RSE of ϕ(*t*) for the forward and backward motions of the robot. An external load (300 g) was placed off-centered at the front of the robot at cycle #50. The average peak values of the instantaneous RSE of the forward and backward motions are shown labeled as “max.” Black arrows show the transient RSE peak of the backward motion. **(B)** Five cycles superimposed and aligned of the PD, and Vn outputs at the end of the experiment [cycles #90-95, black line shown in **(A)** with label B/C]. **(C)** Left and right hemispheres, and the biCNN model firing rates [the same cycles as **(B)**]. **(D)** Control performance in terms of RSE of ϕ(*t*) when the load was placed off-center at the back of the robot [same format as **(A)**]. **(E)** Five cycles superimposed and aligned to the PD and Vn outputs (cycles #90-95, black line shown in **(D)** with label E/F, in the same format as **(B)**. **(F)** Left and right hemispheres, and the biCNN model firing rates (the same cycles as **(E)**, in the same format as **(C)**]. Notation as in Figure 6.

To evaluate the biCNN model during asymmetrical control scenarios, the previous stimulus was repeated. The biCNN model was commanded to follow a sinusoidal [ϕ_*ref*_(*t*) = π sin(2π0.25*t*), θ_*ref*_(*t*) = 0] motion for 100 cycles. In the first asymmetrical condition, the same external load as used in the symmetrical control scenario (300 g, which corresponds to 50% of the robot's mass) was placed on the robot off-center to the front or back from its vertical axis (depicted in Figure 5C) at cycle #50, producing an asymmetrical control scenario for the biCNN model. The second asymmetrical scenario was constructed by changing the angle of the platform on which the robot was moving (see below, depicted in Figure 5D). In the case of the external load, the perturbation was added to the front or back of the robot in two separate experiments (each experiment was repeated three times) to account for the intrinsic differences in the forward and backward motions of the robot. Figure 7 shows that the biCNN model was able to learn to produce the motor commands required for compensating for the first asymmetrical scenario. Figure 7A is the same format as Figure 6A and shows the RSE of ϕ(*t*) for the forward and backward motions of the robot when the load was placed off-center to the front of the robot, demonstrating that forward motion was more affected than backward motion. The backward motion showed a larger average instantaneous peak RSE (2.372 rad) than the forward motion (1.292 rad). However, backward motion rapidly fell below the RSE of the forward motion (Figure 7A, black arrows). The reduction in the forward motion of the robot can be easily observed by comparing the RSE of the forward motion during the symmetrical control scenario (Figure 6A red line, cycles #50-60). In contrast, when the load was placed off-center to the back of the robot (Figure 7D), the backward motion was more affected (average peak RSE 1.023 rad). These results confirm that the conditions in this first control scenario were asymmetrical.

Figures 7A,D also show that the biCNN model was able to adapt and reduce the transitory error produced by the asymmetrical perturbation, so that by the end of the experiment, the RSE values (Figures 7A–D, cycles #90-100) were close to those before the external load was added (Figures 7A–D, cycles #40-45). The outputs of the PD, the Vn, left and right hemispheres, and the biCNN model firing rates (Figures 7B,C,E,F) reflected the external perturbations. During either condition (load to the back or front), the PD output showed large peaks. With respect to the values before the perturbation, the biCNN model increased its output by 79 and 73% when the load was off-center to the back and front, respectively. On the contrary, the average peak-to-peak firing rate of the left hemisphere (Figures 7C,F, red lines), which mainly received error information from the forward motion via *cf*_*left*_, decreased by 16 and 17%, respectively, indicating the preference of this hemisphere to forward motion (a larger reduction caused by LTD). The right hemisphere, which mainly received error information from the forward motion via *cf*_*right*_, decreased by 36 and 28%, showing the opposite preference.

In this first asymmetrical control scenario, the biCNN model was able to account for the asymmetrical condition, despite the change in the dynamics of the control plant. Similar results were found at different frequencies of the desired motion (Supplementary Figure 2). A uni-hemispheric CNN was not able to control the robot (Supplementary Figure 1). In the second asymmetrical control scenario (i.e., using the platform shown in Figure 5D), we further studied the capabilities and generalization of the biCNN model during a more challenging asymmetrical motor control task.

**Figure 8:**
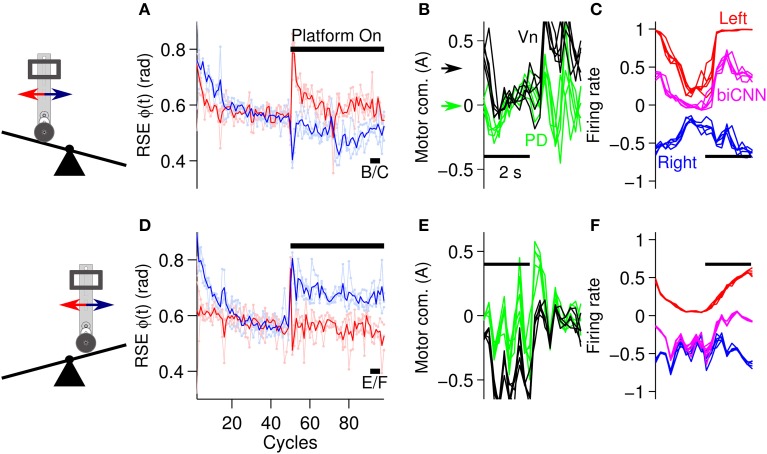
**Performance attained during asymmetrical perturbation control of the robot. (A)** Control performance in terms of RSE of ϕ(*t*) for the forward and backward motions of the robot. The platform was inclined (10 deg) at cycle #50. **(B)** Five cycles are superimposed and aligned to the PD and Vn outputs at the end of the experiment [cycles #90-95, black line shown in **(A)** with label B/C]. The black and green arrows show the DC level of the Vn and PD outputs, respectively. **(C)** Left and right hemispheres, and the biCNN model firing rates [the same cycles as **(B)**]. **(D)** Control performance in terms of RSE of ϕ(*t*) when the platform was declined (−10 deg) [in the same format as **(A)**]. **(E)** Five cycles superimposed and aligned to the PD and Vn outputs at the end of the experiment [cycles #90-95, black line shown in **(D)** with label E/F in the same format as **(B)**]. **(F)** Left and right hemispheres, and biCNN model firing rates [the same cycles as **(E)**, in the same format as **(C)**]. Notation as in Figure 6.

The second asymmetrical scenario consisted of changing the environment of the robot by inclining or declining the platform where the robot moved (Figure 5D). Following the same experimental protocol as in the previous scenarios, the angle of the platform was changed ±10° at cycle #50, and the desired motion was maintained for 100 cycles in total. Figure 8 shows the results in the same format as Figure 7. In general the biCNN model was able to account for inclinations of ±10° by adapting its output. Figures 8A,D show the performance in terms of RSE of ϕ(*t*). There is clear evidence of causation between the motion affected and the asymmetrical conditions, that is, the forward motion was more affected than the backward motion when the robot had to climb the platform in the forward direction (platform inclined), whereas the backward motion was more affected when the robot had to climb in a backward motion (platform declined). This result confirmed our intention of constructing an asymmetrical control scenario for the biCNN model. Figures 8A,D also indicated that the biCNN model adapted and reduced its error in the forward direction (Figure 8A, red lines) to a larger extent than in the backward direction (Figure 8D, blue lines), reflecting once more the intrinsic differences in the mechanics of the robot (also shown in Figure 6). Figures 8B,E show the PD and Vn outputs, and Figures 8C,F show the outputs of the left and right hemispheres and the biCNN model firing rates in the same format as Figures 7B,C. These data demonstrate the active role of the biCNN model in producing the Vn output. When the robot climbed the platform in the forward direction, the Vn output (Figure 8B, black lines and black arrow) produced a positive DC value (approximately 0.3 A) to compensate for the asymmetrical environment. This value was not produced by the PD output because it remained at a zero DC level (Figure 8B, green lines and green arrow) but was produced by the increased DC level of the biCNN model firing rate (Figure 8C, magenta lines). The same behavior occurred when the robot climbed in the backward direction (Figure 8F). Figures 8C,F show the changes in the modulation of the output of the left and right hemispheres (i.e., the average firing rate of the Pk cells) that occurred with the different asymmetrical conditions. The left hemisphere changed from a square-like modulation when the platform was inclined (Figure 8C) to a sinusoidal-like modulation when the platform was declined (Figure 8F). These results demonstrate the asymmetrical adaptation that occurred in the biCNN model to compensate for asymmetrical perturbations to the control plant. Similar results were obtained at different frequencies of the desired motion (Supplementary Figure 2 and Supplementary Movie 1).

### 3.3. Low-frequency spontaneous firing rate in cf is critical for the biCNN model

**Figure 9:**
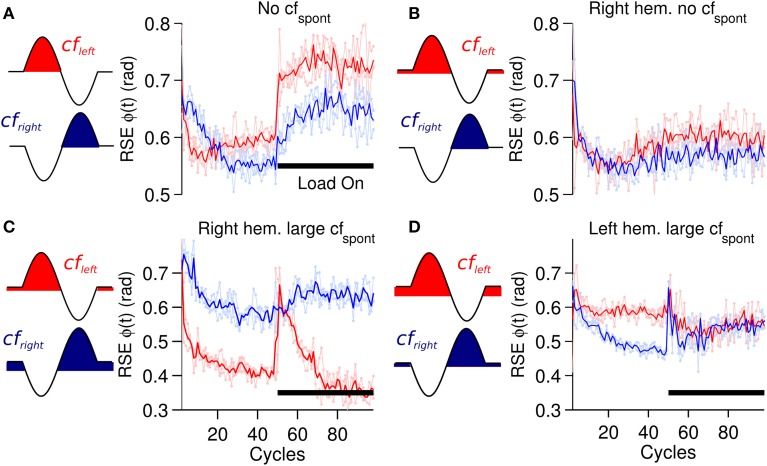
**Performance for forward motion (6 raw performances in light red: average performance in red) and backward motion (6 raw performances in light blue: average performance in blue) in terms of RSE of ϕ(*t*) during different conditions of *cf*_*spont*_. (A)** biCNN model lacking *cf*_*spont*_ during a symmetrical load control scenario. **(B)** biCNN model lacking *cf*_*spont*_ in one hemisphere (right) without external load. **(C)** biCNN model with *cf*_*spont*_ = 0.08 and 0.01 (initial value *cf*_*spont*_ = 0.05) in the left and in the right hemisphere, respectively. **(D)** Opposite combination of *cf*_*spont*_ to that in **(C)**. The cf configuration according to Figure 2 is also included to the left of each figure for reference.

A critical component of the biCNN model is the cf input that drives plasticity at the pf-Pk synapses. The cf input in the current model is sensitive to errors happening in a specific direction and also includes spontaneous (*cf*_*spont*_) activity. The cf input increases its firing rate with motion errors in the preferred direction (ipsidirectional) and decreases its firing rate below the *cf*_*spont*_ to a zero minimum with motor errors occurring in the non-preferred direction (contradirectional) of each hemisphere. To assess the importance of *cf*_*spont*_, we performed a comparison between a cf input lacking spontaneous activity (Figure 2B) and the results obtained thus far. The biCNN model lacking *cf*_*spont*_ was able to control the robot during the close-to-symmetrical control scenario. The control performance in terms of RSE of ϕ(*t*) is shown in Figure 9A in the same format as Figure 6A. However, the biCNN model lacking *cf*_*spont*_ could not decrease the RSE after perturbations during the asymmetrical condition (data not shown). Thus, the performance obtained with the biCNN model lacking the spontaneous firing rate in cf is similar to that obtained with a uni-hemispheric CNN model (Pinzon-Morales and Hirata, in review). These results suggest that the *cf*_*spont*_ is critical for asymmetrical control with the biCNN model. To further evaluate the importance of *cf*_*spont*_, an additional test including the *cf*_*spont*_ in only one of the hemispheres (left) was carried out. The control performance attained in terms of RSE of ϕ(*t*) is shown in Figure 9B. The result, which corresponds to 100 cycles of the sinusoidal desired motion without any external perturbation, shows that the biCNN model initially reduced the RSE (cycle #1-30) but could not sustain this reduction for long. This is because the left hemisphere lacked the contralateral error information in cf and could not change its output to account for inadequate outputs produced by the intact hemisphere, thus endangering the balance of the hemispheres and the overall output of the biCNN model. If such a relationship were true, the balance between the hemispheres and the performance of the model should be tunable by changing the value of the *cf*_*spont*_ in each hemisphere. Figures 9C,D show that this is the case. Figure 9C was obtained by setting the value of *cf*_*spont*_ = 0.08 and 0.01 (the initial value of *cf*_*spont*_ was 0.05) in the right and left hemisphere, respectively. Performance was compromised in the backward direction, which is mainly driven by the right hemisphere receiving the cf with large *cf*_*spont*_. Figure 9D, which was obtained by setting the values in the opposite order, shows the opposite relationship. The performance of the backward direction was affected but the forward direction was severely decreased. Therefore, the cf input, which is direction sensitive in the biCNN model, is critical for asymmetrical control because it balances the contribution of each hemisphere. The information about errors occurring in the non-preferred direction conveyed by the cf input via a reduction of its firing rate below its spontaneous rate proved to be critical for the biCNN model during the asymmetrical control scenario.

## 4. Discussion

We developed a bi-hemispheric neuronal network model of the cerebellum (the biCNN model) that closely mimics anatomical and physiological characteristics of the cerebellar cortex. We included a direction-sensitive climbing fiber (cf) input that encoded sensory error information by altering its spontaneous firing rate to investigate the mechanisms required for asymmetrical motor learning. Our results showed that the bi-hemispheric structure is critical for asymmetrical motor learning, but it also requires a mediator to balance the contribution of the two hemispheres. Here, that role is filled by the cf input, as discussed below.

### 4.1. Cerebellar mechanisms for asymmetrical control

The cerebellar hemispheres are asymmetric in macrostructure and function (Solodkin et al., 2001; Xiang et al., 2003; Hu et al., 2008). The level of asymmetry is subject dependent and has been proposed to be correlated with the level of skill that is required for a particular task (Snyder et al., 1995). Unilateral hand movement tasks without learning components activate the ipsilateral cerebellum, whereas moving the non-dominant hand or complex hand movements are associated with a more bilateral activation pattern in the cerebellum, which supports the suggestion that non-dominant or complex hand movements require more coordinated control from the cerebellum (Jancke et al., 1999; Hu et al., 2008). Cerebellar asymmetries have also been found to correlate with handedness for tool use in apes (Cantalupo et al., 2008). The results of the control engineering experiments carried out here with the biCNN model are in line with this evidence. On one hand, the asymmetrical conditions imposed on the robot demanded the generation of motor commands compensating for the unbalanced dynamics induced in the robot. These motor commands were adequately generated by the biCNN model (Figures 7, 8). On the other hand, control of the two-wheel balancing robot in response to a close-to-symmetrical external perturbation was well handled by the biCNN model (Figure 6) or by uni-hemispheric version (Pinzon-Morales and Hirata, in review). More complex control scenarios, including asymmetrical conditions, were handled only by the biCNN model (Figures 7, 8 and Supplementary Figure 1). Thus, the bi-hemispherical structure reproduced a form of asymmetrical motor learning observed in the real cerebellum, and also proved to be critical for compensating for complex control tasks (i.e., asymmetrical control conditions) during our control engineering experiments.

Lesioning of the cerebellar hemispheres compromises both the ipsilateral and contralateral motor performance. Monkey experiments have shown that the speed of saccadic eye movements was affected in the ipsiversive and contraversive directions when one cerebellar hemisphere (H-VII) was lesioned (Ohki et al., 2009). Unilateral cerebellar hemisphere infarction in humans also significantly reduced ipsilateral saccadic adaptation (Choi et al., 2008). The results presented here demonstrate that the control performance in the forward and backward motion of the robot, which was directly related with the left and right hemisphere outputs, respectively, were affected not only when the asymmetrical perturbation was added to the ipsidirectional side of the robot but also (albeit in a lesser degree) when the perturbations were located on the contradirectional side of the robot (Figures 7A,D, 8, red and blue lines). Furthermore, the results of the present model suggest a mechanism that explains the interaction observed between the cerebellar hemispheres. This mechanism involves the cf input, which drove the plasticity at pf-Pk synapses in each hemisphere. This input increased its firing rate above its spontaneous firing rate with ipsidirectional erroneous motions, whereas it reduced its firing rate with contradirectional erroneous motions. This configuration of cf input has been suggested in monkey experiments, where complex spike activity of Pk cells is highly correlated with cf activity. In the monkey, cf input conveys direction-sensitive motor error by increasing its firing rate and also information of the non-preferred direction by reducing its firing rate or presenting firing pauses during horizontal VOR adaptation (Hirata et al., 2006, 2007). Our results reinforce the relevance of this configuration of the cf input (Figure 9). The cf worked as a differential link that balanced the contribution of each hemisphere to the overall input to the Vn. Removing or adjusting the spontaneous firing rate of the cf resulted in a reduction of motor performance or completely abolished the system's ability to compensate for an asymmetrical perturbation of the robot. Therefore, a bi-hemispheric structure with direction selective cf input and adequate spontaneous cf activity is critical for cerebellar asymmetrical motor learning.

### 4.2. Limitation and generalization of the cerebellar model

In the present cerebellar model, spike patterns or temporal effects were impossible to evaluate due to the level of abstraction. Such an evaluation would require the construction of a cerebellar network with spiking neuronal models, which would prevent the real-time real-world application of a model of the size employed here (9 k neurons and more than 200 k synapses). However, the present model was constructed following spatially consistent features of a 100 μm sided cube of cerebellar cortex. Spatial behaviors such as the center-surround filtering property of the granular layer that have been reported in a similar computational model (Solinas et al., 2010) were not evaluated in this experiment. These features may be explored in a future work. In addition, our model included distributed plasticity at two loci, pf-Pk, and Pk-Vn synapses. The latter have been proposed to be the location where motor learning is stored after consolidation (Masuda and Amari, 2008), whereas the former are regarded as the locus of fast adaptation in the cerebellum (Kassardjian et al., 2005; Ito, 2011). The pf-Pk synapses in the biCNN model exhibited faster learning and convergence than the Pk-Vn synapses (data not shown), suggesting that both loci supported the overall plasticity in the model but in different timescales. Other sites of plasticity in the cerebellum and their involvement in motor learning have been argued (McElvain et al., 2010; Gao et al., 2012; Garrido Alcazar et al., 2013), such as the synapses between the molecular layer interneurons and Pk cells. Including other sites of plasticity remains a future improvement to our model.

The information carried by the cf input to the biCNN model, which in the present document was sensory error in kinematics units (Hirata et al., 2002; Ito, 2011, 2013; Pinzon-Morales and Hirata, in review), has also been suggested to be encoded in motor coordinates in dynamic units (Kawato, 1999; Ito, 2013). We tested our model with a cf input carrying motor error information in different control plants (Pinzon-Morales and Hirata, in review) and control scenarios (symmetrical and asymmetrical loads, data not shown). The results obtained were similar to those reported here. Using sensory error slightly improved the motor performance achieved, although both configurations of cf achieved stable control of the plant, regardless of the control conditions tested (a discussion about the origin and information in cf has been presented elsewhere (Pinzon-Morales and Hirata, in review). Regarding the control plant, the two-wheeled inverted pendulum was employed here because it is one of the most challenging plants commonly used to test control strategies (Li et al., 2013). We believe that the biCNN model can be successfully employed in other control plans to solve real-life engineering problems. As examples, virtual reality control simulations with our biCNN model of several control objects, including a direct current motor and a quadcopter have been added in the repository containing the biCNN model and is freely available for downloading.

### Conflict of interest statement

The authors declare that the research was conducted in the absence of any commercial or financial relationships that could be construed as a potential conflict of interest.

## Supplementary material

Supplementary Figure 1 **Asymmetrical control scenarios with a uni-hemispheric CNN (Pinzon-Morales and Hirata, in review). (A)** Control performance in terms of RSE of ϕ(*t*) for the forward (red line, and light red lines) and backward (blue line and light blue lines) motion of the robot (*N* = 6) (left panel). The desired and yielded motions of the wheels of the robot [i.e., ϕ_*ref*_(*t*) and ϕ(*t*)] are shown in black and red lines (right panel), respectively. **(B)** Asymmetrical control scenario with the inclined platform (10 deg) at cycle #50. In the same format as **(A)**.

Supplementary Figure 2 **Comparison of the performance achieved during control of the robot with the biCNN model at different frequencies of the desired motion and different perturbations (symmetrical and asymmetrical). (A)** Baseline performance obtained when using only the PD controller. Average control performance (total number of repetitions of the experiment *N* = 6) and raw performances in terms of RSE of ϕ(*t*) for the forward (red line, and light red lines) and backward (blue line and light blue lines) motion of the robot. The frequency of the stimulus ranged from 0.2 to 0.3 Hz as indicated in each figure. **(B)** Performance during the close-to-symmetrical control scenario. **(C)** Performance during the asymmetrical scenario (load to the front). **(D)** Performance during the asymmetrical scenario (load to the back). **(E)** Performance when the platform where the robot moved was inclined 10°. **(F)** Performance when the platform was declined 10°.
